# Clarity and Confusion in the Development of Youth Aerobic Fitness

**DOI:** 10.3389/fphys.2019.00979

**Published:** 2019-07-30

**Authors:** Neil Armstrong, Jo Welsman

**Affiliations:** Children’s Health and Exercise Research Centre, University of Exeter, Exeter, United Kingdom

**Keywords:** aerobic fitness, assessment, clinical red flags, fat free mass, multilevel allometric modeling, peak oxygen uptake, scaling, youth

## Abstract

Peak oxygen uptake ($\dot{V}O_{2}$) is internationally recognized as the criterion measure of youth aerobic fitness, but flawed laboratory assessments and fallacious interpretations of peak $\dot{V}O_{2}$ in ratio with body mass have confused our understanding of the development of aerobic fitness. Moreover, the recent emergence of specious predictions of peak $\dot{V}O_{2}$ from performance tests and the promotion of spurious “clinical red flags” and cardiometabolic cut-points have confused our understanding of the relationship between youth aerobic fitness and health. Recent longitudinal studies of 10–18-year-olds using multilevel allometric modeling have empirically demonstrated that peak $\dot{V}O_{2}$ increases in accord with sex-specific, concurrent changes in age- and maturity status-driven morphological covariates with the timing and tempo of changes specific to individuals. During both cycle ergometry and treadmill running age- and maturity status- driven changes in fat free mass have been revealed as the most powerful morphological influences on the development of youth aerobic fitness. To bring some clarity to current confusion, this paper argues that future studies must be founded on rigorous assessment and interpretation of peak $\dot{V}O_{2}$ and ensure that they address the development of youth aerobic fitness and its relationship with present and future health in relation to appropriate sex-specific morphological covariates governed by individual biological clocks.

## Introduction

Aerobic fitness defines the ability to deliver oxygen from the atmosphere to the skeletal muscles and to use it to generate energy to support muscle activity during exercise. Peak oxygen uptake ($\dot{V}O_{2}$), the highest rate of oxygen consumed during an incremental exercise test to exhaustion, limits the capacity to perform aerobic exercise and is internationally recognized as the best single measure of youth aerobic fitness. Peak $\dot{V}O_{2}$ is the most researched physiological variable in pediatric exercise physiology but understanding of the development of youth aerobic fitness is embedded in confusion with shoddy assessments and fallacious interpretations of peak $\dot{V}O_{2}$ during growth and maturation. Moreover, youth aerobic fitness and its relationship with current and future health is shrouded in confusion through a resurgence of specious predictions of peak $\dot{V}O_{2}$ from performance tests and the promotion of spurious “cardiometabolic cut-points” and “clinical red flags.” To bring some clarity to current confusion, this mini-review critically reviews the evidence relating peak $\dot{V}O_{2}$ to changes in age, maturity status, body size, and body composition with reference to health.

## Confusion in the Development of Youth Aerobic Fitness

### Assessment

Antoine-Laurent Lavoisier was the first to experiment with the measurement of $\dot{V}O_{2}$ during exercise in the 1770s, but it is Hill and Lupton (1923), who introduced the concept of a near-linear relationship between $\dot{V}O_{2}$ and running speed until, despite an increase in running speed, a plateau in $\dot{V}O_{2}$ emerges at the point of maximal $\dot{V}O_{2}$. In the first laboratory-based study of boys Robinson (1938) ran 6–17-year-olds on a treadmill at a speed of 7 miles⋅h^–1^ up an 8.6% gradient until they were “exhausted” and reported their $\dot{V}O_{2}$ as “maximal.” In his seminal study of boys and girls, Åstrand (1952) criticized Robinson’s (1938) methodology and adopted a more rigorous discontinuous, incremental exercise protocol over several days. He noted that the $\dot{V}O_{2}$ plateau proposed by Hill and Lupton (1923) was found in only 50% of schoolchildren. This phenomenon was subsequently confirmed in large studies with both pre-pubertal (Armstrong et al., 1995) and pubertal (Armstrong et al., 1991) youth but generally ignored for decades. When it was addressed and the term peak $\dot{V}O_{2}$ introduced (Armstrong and Davies, 1981) scientific journals confused understanding of youth aerobic fitness for several years by often rejecting papers reporting peak $\dot{V}O_{2}$ on the basis that maximal values were not attained.

Peak $\dot{V}O_{2}$ is now recognized as the “gold standard” measure of youth aerobic fitness but researchers continue to wrestle with factors related to its rigorous determination. In cardiopulmonary exercise tests children and adolescents normally exercise to voluntary exhaustion but there is no way to confirm, in the single tests typical of most studies, whether an individual has delivered a maximal effort. The experience of the testing team, supported by subjective criteria of intense effort (e.g., facial flushing, sweating, hyperpnoea, and unsteady gait), is critical in deciding whether a maximal value has been attained. However, to verify efforts as maximal, secondary criteria such as pre-set (and often submaximal) values of heart rate (HR), respiratory exchange ratio, and blood lactate accumulation at the termination of exercise are widely used. However, all secondary criteria exhibit large individual variations and are exercise protocol and ergometer dependent (Armstrong and McManus, 2017). The growing tendency to rely on what are clearly submaximal criteria such as HR ≥ 85% of predicted maximum to confirm maximal values has confused understanding of both the development of aerobic fitness and its purported relationship with other health-related variables. Barker et al. (2011) reported that terminating a test with secondary criteria can underestimate a child’s “true” peak $\dot{V}O_{2}$ by ∼10–22%.

Both treadmills and cycle ergometers are used routinely in pediatric exercise laboratories. Due to the greater muscle mass, enhanced venous return, higher stroke volume (SV), and reduced peripheral resistance during running, mean treadmill-determined values are ∼11–14% higher than those determined on a cycle ergometer. Ergometer-dependent differences in peak $\dot{V}O_{2}$ at specific ages vary with sex, age, maturity status, and morphological covariates (Armstrong and Welsman, 2019a) but some reviewers have confused understanding of the development of aerobic fitness by combining treadmill- and cycle ergometer-determined peak $\dot{V}O_{2}$ values (e.g., Bar-Or and Rowland, 2004). Other authors have added to the confusion by “correcting” for ergometer differences by multiplying cycle ergometer values by fixed percentages regardless of sex, age, or maturity-status (e.g., Stavnsbo et al., 2018) or assuming that increasing cycle ergometer values “*by ∼2–3 mL⋅kg^–^^1^⋅min^–1^ would make them equivalent to values obtained by a treadmill protocol*” (Aadland et al., 2019, p. 248).

### Development

Robinson (1938) initially reported boys’ “maximal” $\dot{V}O_{2}$ in L⋅min^–1^ before “*referring them to body weight*” (p. 280), analyzing his data in ratio with body mass (i.e., in mL⋅kg^–^^1^⋅min^–1^), and initiating an approach for “controlling” for growth that has confused pediatric exercise physiology for over 80 years.

Tanner (1949) unequivocally established that expressing peak $\dot{V}O_{2}$ in ratio with body mass was fallacious. Subsequent reviews have explained the statistical assumptions underlying ratio scaling of peak $\dot{V}O_{2}$ with body mass and demonstrated that they are seldom (if ever) met (Welsman and Armstrong, 2008, 2019). Yet the vast majority of pediatric exercise studies still interpret peak $\dot{V}O_{2}$ in ratio with body mass and reports of spurious correlations with indicators of cardiovascular health are common (Mintjens et al., 2018). Purported relationships between ratio-scaled peak $\dot{V}O_{2}$ and other health-related variables have confused the association of youth aerobic fitness with current and future health. For example, any relationship between cardiovascular risk factors in overweight/obese youth with ratio-scaled peak $\dot{V}O_{2}$ is more likely to reflect overweight/obese status than aerobic fitness (Loftin et al., 2016).

In practice, ratio-scaled peak $\dot{V}O_{2}$ favors lighter (e.g., clinically underweight or delayed maturing) youth and penalizes heavier (e.g., overweight or advanced maturing) youth. Literature reviews (e.g., Krahenbuhl et al., 1985) and textbooks (e.g., Bar-Or and Rowland, 2004) reporting ratio-scaled peak $\dot{V}O_{2}$ have confused understanding of developmental exercise physiology for decades. For example, peak $\dot{V}O_{2}$ data ratio-scaled with body mass indicates that boys’ aerobic fitness decreases slightly or remains unchanged from 10 to 18 years, whilst in girls a progressive decline is apparent over the same time scale. However, when body mass is appropriately controlled using log-linear regression boys’ peak $\dot{V}O_{2}$ increases with age and girls’ peak $\dot{V}O_{2}$ increases at least until 13 or 14 years and then levels-off (Welsman et al., 1996). Similarly, ratio-scaled data indicate that once body mass is controlled for maturity status has no effect on peak $\dot{V}O_{2}$ (e.g., Fahey et al., 1979) whereas allometric (log-linear regression) scaling has demonstrated positive effects of maturity status in addition to those of age and body mass in both boys and girls (Armstrong et al., 1998).

### Performance Tests and Health-Related Cut-Points

A recent resurgence of interest in estimating/predicting peak $\dot{V}O_{2}$ from 20-m shuttle run test (20mSRT) performance, has confused understanding of youth aerobic fitness (Armstrong and Welsman, 2018; Welsman, 2019). The 20mSRT is not a measure of aerobic fitness but a function of willingness to run between two lines 20 m apart whilst keeping pace with audio signals which require the running speed to increase each minute until participants are unwilling or unable to maintain the pace. The number of shuttles/stages completed is converted into an estimate of peak $\dot{V}O_{2}$ in ratio with body mass through one of at least 17 published prediction equations (Tomkinson et al., 2017). A recent meta-analysis reported that over half of published correlation coefficients between 20mSRT scores and “true” peak $\dot{V}O_{2}$ explain less than 50% of the total variance in peak $\dot{V}O_{2}$ and concluded, “*testers must be aware that the performance score of the 20MSR test is simply estimation and not a direct measure of cardiorespiratory fitness*” (Mayorga-Vega et al., 2015, p. 545). The capacity for confusion created by uncritical application of 20mSRT data to relationships with indicators of health is revealed by the 95% range for a “true” peak $\dot{V}O_{2}$value estimated from 20mSRT performance being ∼10 mL⋅kg^–^^1^⋅min^–1^ or ∼24% (Tomkinson et al., 2019a).

Further confusion has arisen over the introduction and promotion of “cardiometabolic cut-points” “*to define children with poor cardiometabolic health*” (Aadland et al., 2019, p. 240) and “clinical red flags” to identify “*children and adolescents who may benefit from primary and secondary cardiovascular prevention programming*” (Ruiz et al., 2016, p. 1451). The validity of these “cut points” and “clinical red flags,” both based on ratio-scaled peak $\dot{V}O_{2}$, is also challenged through them being derived from combined cycle ergometer- and treadmill-determined peak $\dot{V}O_{2}$values with a fixed 5% added to cycle ergometer values (Stavnsbo et al., 2018); an amalgam of data from treadmill- and cycle ergometer (+5%)-determined peak $\dot{V}O_{2}$ and peak $\dot{V}O_{2}$ predicted from 20mSRTs (Aadland et al., 2019); and solely from 20mSRT predictions of peak $\dot{V}O_{2}$ (Ruiz et al., 2016). None of the proposed “cut points” consider maturity status. “Clinical red flags” also take no account of age with the indefensible assumption that a pre-pubertal 8-year-old is comparable to a post-pubertal 18-year-old with the same peak $\dot{V}O_{2}$ ratio-scaled with body mass.

## Clarity in the Development of Youth Aerobic Fitness

### Assessment

The laboratory measurement of peak $\dot{V}O_{2}$ has been progressively developed and refined as new technology has replaced the classic Douglas bag method initially with mixing chambers and more recently with breath-by-breath analyses. The importance of appropriate exercise test protocols, ergometers, breathing interfaces, size of components of respiratory gas collection systems, and sampling intervals during growth and maturation cannot be overemphasized and all methodology, apparatus, and calibration techniques should be carefully reported (see McManus and Armstrong, 2017; Falk and Dotan, 2019 for comprehensive reviews).

The typical error of youth peak $\dot{V}O_{2}$ rigorously determined in three tests each a week apart is in our hands ∼4% (Welsman et al., 2005). To increase confidence in obtaining a “true” peak $\dot{V}O_{2}$ in a single session an initial exercise test can be confirmed with a validation test. For example, a cycle ergometer ramp test to exhaustion followed ∼15 min later by a validation test consisting of a 2 min warm-up before a step change to 105% of the peak power elicited at the end of the initial test. On the few occasions (in our hands <5%) that the peak $\dot{V}O_{2}$is higher than in the initial test the validation test can be repeated at 110% of peak power following full recovery (Barker et al., 2011). This protocol is facilitated by children’s ability to recover from heavy exercise faster than adults (Ratel et al., 2006; Armstrong, 2019).

### Development

Youth peak $\dot{V}O_{2}$ has been comprehensively documented but flawed experimental designs, statistical analyses, and data interpretation have limited insights into the development of aerobic fitness. Use of equipment and exercise protocols designed for adults, small sample sizes, combining of data from boys and girls, only reporting peak $\dot{V}O_{2}$ ratio-scaled with body mass, and serious concerns over whether true maximal values have been attained make peak $\dot{V}O_{2}$ data from young children difficult to interpret (Armstrong and Welsman, 1994). The focus herein will therefore be on the more secure database of 10–18-year-olds.

**FIGURE 1:**
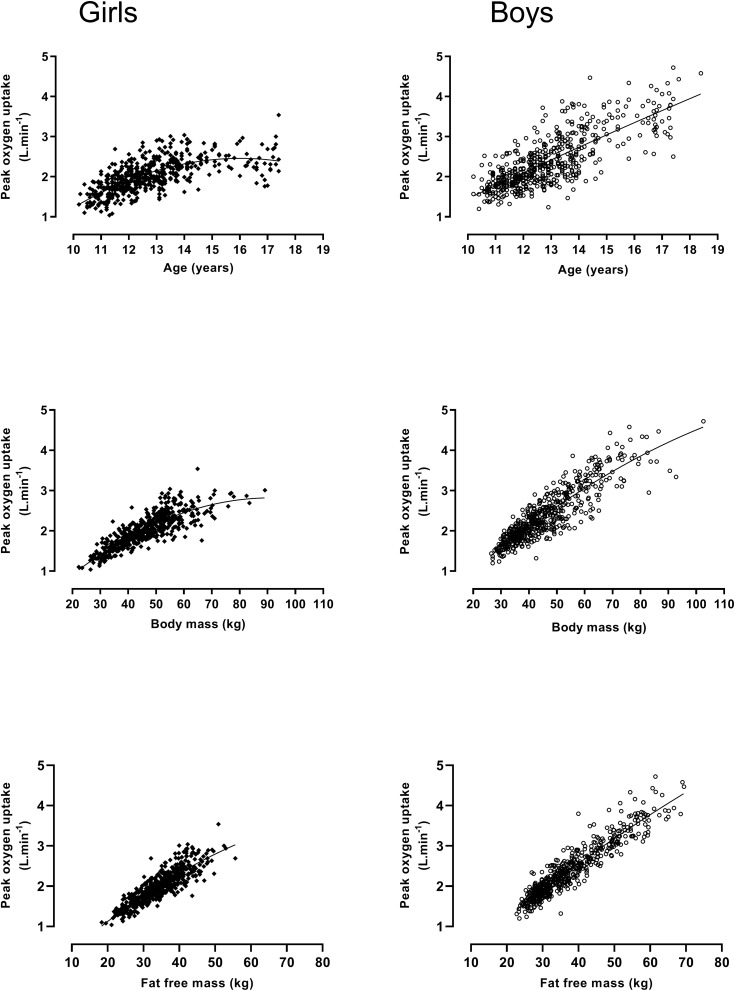
Peak oxygen uptake by age, body mass, and fat free mass in 10–18-year-old girls and boys: Figure founded on 1057 determinations of peak oxygen uptake, age, body mass, and fat free mass; girls (*n* = 501), boys (*n* = 556). Data from Armstrong and Welsman (2019b).

The snapshot moments in time reflected by cross-sectional studies provide few insights and rigorous examination of the development of aerobic fitness requires longitudinal studies (Armstrong and McManus, 2017). Longitudinal data based on 1057 treadmill determinations of peak $\dot{V}O_{2}$ have revealed that aerobic fitness is significantly correlated with age (*r* = 0.78, boys and 0.64, girls), body mass (*r* = 0.89, boys and 0.83, girls), and fat-free mass (FFM) (*r* = 0.94, boys and 0.87, girls) (Armstrong and Welsman, 2019b). As can be seen in Figure 1, there is a near-linear increase in boys’ peak $\dot{V}O_{2}$ from 10 to 18 years. In girls, a near-linear increase in peak $\dot{V}O_{2}$ until ∼13–14 years of age is followed by a leveling-off from ∼14 to 18 years. Boys’ peak $\dot{V}O_{2}$ almost doubles from 10 to 18 years while girls’ values increase by ∼50% over the same time period. Pre-pubertal boys’ peak $\dot{V}O_{2}$ values are, on average, ∼12% higher than those of similar aged pre-pubertal girls and the mean sex difference in peak $\dot{V}O_{2}$ increases as young people progress through adolescence reaching ∼50% in post-pubertal 18-year-olds. Figure 1 illustrates the relationships of body mass and FFM with peak $\dot{V}O_{2}$ with sex differences evident throughout the age range. Girls’ peak $\dot{V}O_{2}$ in relation with body mass tends to taper-off from ∼60 kg. Body mass includes both fat mass which is metabolically inert (Goran et al., 2000) and FFM which reflects the active muscle. The relationship of peak $\dot{V}O_{2}$ with FFM (estimated from the equations of Slaughter et al., 1988) is remarkably linear from 10 to 18 years in both sexes (Armstrong and Welsman, 2019b).

In a parallel longitudinal study 72 boys and 64 girls also had their peak $\dot{V}O_{2}$ determined on a cycle ergometer. The pattern of peak $\dot{V}O_{2}$ relationships with age, body mass, and FFM were similar to those on a treadmill although the magnitude of the covariates varied. Mean treadmill values of peak $\dot{V}O_{2}$ were significantly (*p* < 0.05) higher in boys on each testing occasion but the percentage difference varied with age, peaking at 13 years. Moreover, some individuals in both sexes demonstrated higher values on a cycle ergometer than a treadmill on at least one test occasion further illustrating the folly of predicting treadmill-determined peak $\dot{V}O_{2}$ by adding a fixed percentage to cycle ergometer values regardless of age, sex, and maturity status (Armstrong and Welsman, 2019a).

Collectively these longitudinal data also unequivocally show the fallacy of ratio scaling peak $\dot{V}O_{2}$ with body mass, regardless of whether it is determined during mass-supporting or mass- supported exercise. Statistical assumptions underlying ratio scaling include a perfect correlation (i.e., *r* = 1.0) between peak $\dot{V}O_{2}$ and body mass which was not met and an allometric exponent of 1.0 for body mass, a value which fell outside the 95% confidence limits. Moreover, significant negative correlations between ratio-scaled peak $\dot{V}O_{2}$ and body mass demonstrated that body mass was not controlled for by ratio scaling (Armstrong and Welsman, 2019a,b).

The application of multilevel regression modeling to trained (Nevill et al., 1998) and untrained youth (Armstrong et al., 1999) and the technique’s on-going refinement (Rasbash et al., 2018) has enabled multiple, individual growth trajectories to be examined in relation to the development of aerobic fitness. The effects of sex, age, maturity status, body size, and body composition on peak $\dot{V}O_{2}$can be partitioned concurrently within an allometric framework to provide a sensitive interpretation of youth aerobic fitness. Armstrong and Welsman (2019a, b) adopted this approach and explored the development of peak $\dot{V}O_{2}$ through a series of models which are shown in Table 1. In contrast to traditional ratio-scaled interpretations, the initial models 1.1 (girls) and 2.1 (boys) show that with body mass controlled for, peak $\dot{V}O_{2}$ increases with age in both sexes. The negative age^2^ term indicates that the size of the age effect reduces as the rate of growth decreases. Stature was not a significant (*p* > 0.05) covariate in any of the models. In conflict with ratio-scaled data, the addition of maturity status, in the form of the stages of pubic hair described by Tanner (1962), showed each stage to have a positive effect on peak $\dot{V}O_{2}$ independent of those from body mass and age (models 1.2 and 2.2). The introduction of sum of triceps and subscapular skinfolds to act with body mass as a surrogate of FFM (Roemmich et al., 1997) resulted in the effects of maturity status being negated but age and age^2^ remained significant covariates. These models (1.3 and 2.3) were the best statistical fit (*p* < 0.05) of the data and demonstrate the powerful effects of FFM on the development of aerobic fitness.

**TABLE 1:** Multilevel regression models for peak oxygen uptake.

	**Girls model 1.1**	**Girls model 1.2**	**Girls model 1.3**	**Boys model 2.1**	**Boys model 2.2**	**Boys model 2.3**
	
	**Log_*e*_ peak oxygen uptake**	**Log_*e*_ peak oxygen uptake**	**Log_*e*_ peak oxygen uptake**	**Log_*e*_ peak oxygen uptake**	**Log_*e*_ peak oxygen uptake**	**Log_*e*_ peak oxygen uptake**
Fixed part						
Constant	−1.701 (0.119)	−1.657 (0.127)	−2.004 (0.117)	−1.861 (0.121)	−1.694 (0.123)	−2.273 (0.099)
Log_*e*_ body mass	0.631 (0.031)	0.609 (0.034)	0.815 (0.038)	0.713 (0.032)	0.655 (0.033)	0.964 (0.031)
Age	0.035 (0.004)	0.024 (0.006)	0.020 (0.005)	0.051 (0.005)	0.031 (0.005)	0.023 (0.004)
Age^2^	−0.010 (0.001)	−0.008 (0.001)	−0.007 (0.001)	−0.004 (0.001)	ns	−0.003 (0.001)
Pubic hair 2	–	0.038 (0.013)	ns	–	0.030 (0.011)	ns
Pubic hair 3	–	0.046 (0.015)	ns	–	0.063 (0.013)	ns
Pubic hair 4	–	0.052 (0.018)	ns	–	0.091 (0.015)	ns
Pubic hair 5	–	0.055 (0.023)	ns	–	0.091 (0.023)	ns
Log_*e*_ skinfolds	–	–	−0.129 (0.018)	–	–	−0.185 (0.013)
Random part						
Level:2						
Var (cons)	0.006 (0.001)	0.006 (0.001)	0.004 (0.001)	0.007 (0.001)	0.006 (0.001)	0.003 (0.000)
Level: 1						
Var (cons)	0.004 (0.000)	0.004 (0.000)	0.004 (0.000)	0.005 (0.000)	0.004 (0.001)	0.004 (0.000)
−2 × loglikelihood	−1060.443	−951.197	−1107.768	−1088.073	−1028.937	−1238.092

*Vales are model estimates (standard error); boys’ data centered on mean age 12.9 years; girls’ data centered on mean age 12.8 years; ns, not significant (*p* > 0.05); –, not entered; Table founded on 1057 determinations of peak oxygen uptake (boys, 556; girls, 501) and 972 (boys, 516; girls, 456) assessments of maturity status. Data from Armstrong and Welsman (2019b).*

Boys’ FFM increases, on average, by ∼90% from 11 to 16 years. It is, however, maturation which drives changes in FFM. This is evidenced by percentage changes in FFM being at their zenith around the time of peak height velocity (PHV) when FFM increases by ∼83% over the period 2 years pre-PHV to 2 years post-PHV. Girls’ FFM increases by ∼40% over the same age range with the greatest increase (∼31%) occurring over a 2 year period centered on PHV, before it levels-off in accord with the development of peak $\dot{V}O_{2}$ (Armstrong, 2019; Baxter-Jones et al., 2003).

For ethical and technological reasons the physiological mechanisms underpinning the development of youth aerobic fitness remain to be fully elucidated (Warburton and Bredin, 2017). HR at peak $\dot{V}O_{2}$is independent of sex, age, maturity status, body size, and body composition at least until the late teens (Rowland, 2017). Developmental changes in peak $\dot{V}O_{2}$ are therefore a function of increases in SV and/or arterio-venous oxygen difference. Both oxygen delivery and oxygen utilization are facilitated by increases in FFM (Armstrong and McManus, 2017). Data on the development of arterio-venous oxygen difference are not available but the positive effects of FFM on the longitudinal development of SV has been demonstrated with multilevel allometric modeling (Armstrong and Welsman, 2002). Moreover, it has been reported that sex differences in SV disappear when it is expressed relative to allometrically scaled FFM (Vinet et al., 2003).

### Performance Tests and Health-Related Cut-Points

The evidence base outlined herein shows that is simplistic to describe aerobic fitness in a cross-sectional snapshot in relation to a single morphological covariate. Analyses of the development of aerobic fitness must take account not only of age but also of sex-specific, maturation-driven changes in FFM which are governed by individual biological clocks. 20mSRT predictions of peak $\dot{V}O_{2}$ have confused our understanding of youth aerobic fitness with proposals to establish international age-related norms (Tomkinson et al., 2019b), to provide “reference standards” for children as young as 2 years (Cadenas-Sanchez et al., 2019), to initiate fitness surveillance programs (Lang et al., 2018), and to promote inter-country comparisons of “who are the fittest?” (Lang et al., 2016). Moreover, youth who raise a “clinical red flag” or cross an age-related “cardiometabolic cut point” are more likely to be suffering from what Tanner (1949) identified as, “*no more formidable a disease than statistical artifact*” (p. 3) than warranting medical attention.

## Conclusion

Peak $\dot{V}O_{2}$ increases in accord with sex-specific, concurrent changes in age- and maturity status-driven morphological covariates with the timing and tempo of changes specific to individuals. For clarity future studies should ensure that they address youth aerobic fitness and its relationship with present and future health with this firmly in mind.

## Author Contributions

Both authors conceived the review, drafted the manuscript, and reviewed and approved the final version of the manuscript.

## Conflict of Interest Statement

The authors declare that the research was conducted in the absence of any commercial or financial relationships that could be construed as a potential conflict of interest.
